# How does vertical integration promote innovation corporate social responsibility (ICSR) in the coal industry? A multiple-step multiple mediator model

**DOI:** 10.1371/journal.pone.0217250

**Published:** 2019-06-12

**Authors:** Ziyuan Sun, Yanli Li, Man Wang, Xiaoping Wang, Yiwen Pan, Feng Dong

**Affiliations:** School of Management, China University of Mining and Technology, Xuzhou, China; The Bucharest University of Economic Studies, ROMANIA

## Abstract

This paper aims to demonstrate the impact of coal enterprises’ vertical integration on the performance of innovation corporate social responsibility (ICSR) and to elaborate its specific transmission path through financing structure and market power. This paper takes the data of A-share listed coal company from 2008 to 2017 as samples, uses input-output table method to measure the degree of vertical integration, and explores the relationship between the four factors through multiple linear regression and Bootstrap method. We found that: (1) the vertical integration of coal enterprises has positive incentives for the promotion of ICSR. (2) Financing structure and market power play a chain intermediary role in this process of incentive. (3) In areas with high marketization process, vertical integration has more significant incentive effect on ICSR. The paper extends the research on the relationship between vertical integration and innovation, which provides a reference for the improvement of China’s supply-side reform and corporate social responsibility (CSR) fulfillment policy. It is helpful to promote the sustainable development of the coal industry, stimulate the innovation vitality of enterprises, and improve the fulfillment of CSR.

## Introduction

**Fig 1:**
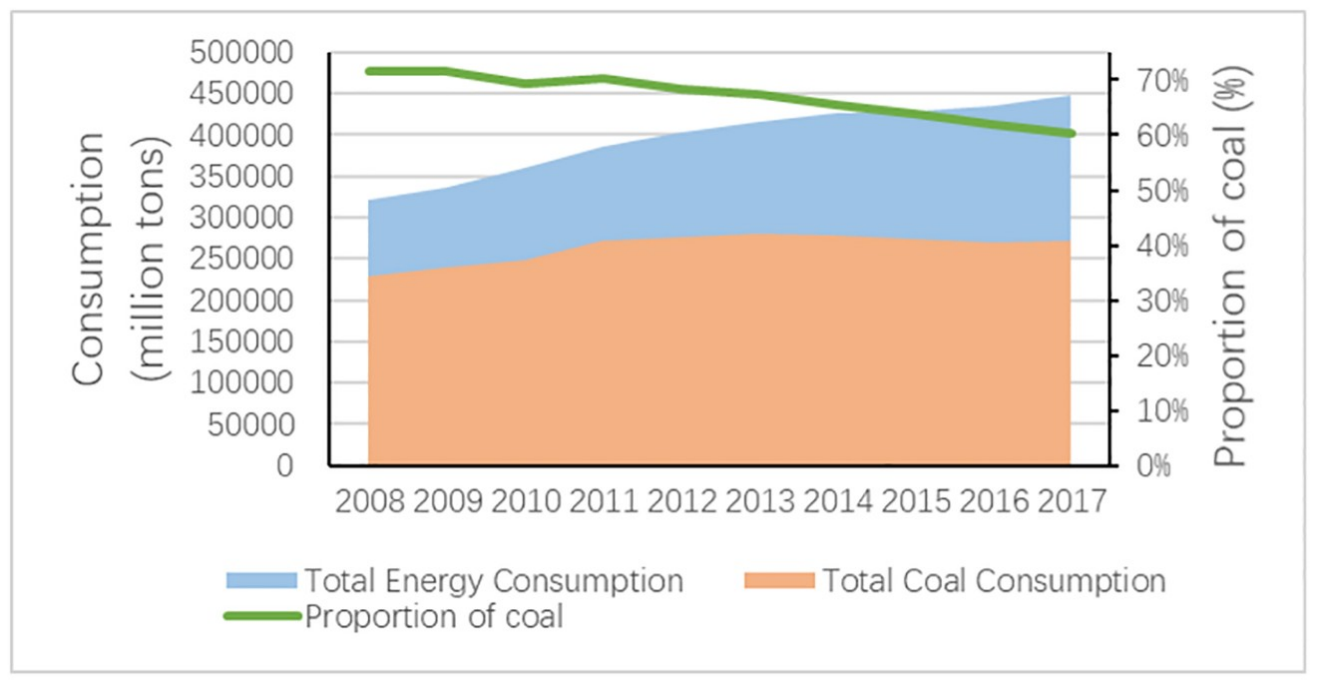
Total coal consumption and its proportion in total energy consumption in China, 2008-2017.

With the increasingly fierce competition in the global low-carbon economy, it has been more and more essential to improve energy efficiency and promote the sustainable development of the resource industry while ensuring the interests of all people. Fossil resources play an important role in China’s economic and social development. The energy structure of “rich coal, deficient oil, and lean gas” [1] determines that China is still the largest coal producer and consumer in the world. According to the statistical yearbook issued by China’s national bureau of statistics, China’s coal production and consumption accounted for 45.6% and 46.4% of the world in 2017, respectively. Among them, coal consumption accounted for 60.4% of China’s primary energy consumption structure, resulting in fossil energy accounting for 82.3% of China’s primary energy production structure. Although coal consumption in China has a downward trend, it still maintains about 59% of China’s total energy consumption in 2018 [2](Fig 1). Coal combustion produces a large amount of wastewater, waste gas, and solid waste every year. Therefore, as a kind of high pollution and high emission non-clean energy [3], coal must shoulder the social responsibility of protecting the environment. The original extensive management mode of China’s coal industry needs to be further optimized.

The task of an enterprise is not only to develop and make profits, but also to assume responsibility for employees, consumers, communities, and the environment. Social responsibility management has become one of the core tasks of enterprise management. Corporate social responsibility (CSR) means that while creating profits and benefiting shareholders and employees, enterprises must also pay attention to external factors such as consumers, communities, and the environment [4]. Its core is to protect the legitimate rights and interests of stakeholders. CSR is a concept with broad connotations, which includes the responsibilities of enterprises to the whole society, including health, safety, and environmental protection. Obviously, it also includes the promotion of economic and social transformation, national technological innovation, and other broader content. This paper focuses on innovation corporate social responsibility (ICSR). It means that enterprises recognize that high-quality and responsible technological innovation actions can guide sustainable development and business success, and independently induce scientific and technological progress through technological research in the relationship with stakeholders, thus contributing to economic, social development and environmental protection. ICSR incorporates social and environmental concerns into business operations. That is to say, ICSR is not a series of activities added to the daily business activities of enterprises. Instead, ICSR should be just a question of how enterprises innovate and develop: R&D investment, patent registration, patent protection, patent trial, the industrialization of scientific and technological achievements, etc. By increasing investment in innovation, enterprises develop high-quality invention patents and create new products, services, or processes. While gaining its own economic or other benefits, it also brings impetus to the improvement of people’s quality of life through the renewal of technology and products. It is an important manifestation of CSR to develop high-quality invention patents that are in line with the national development strategy and can provide better products and improve service quality. Therefore, one of the most important CSR is to develop high-quality invention patents and offer better products, services, or processes to make society better and benefit the public.

**Fig 2:**
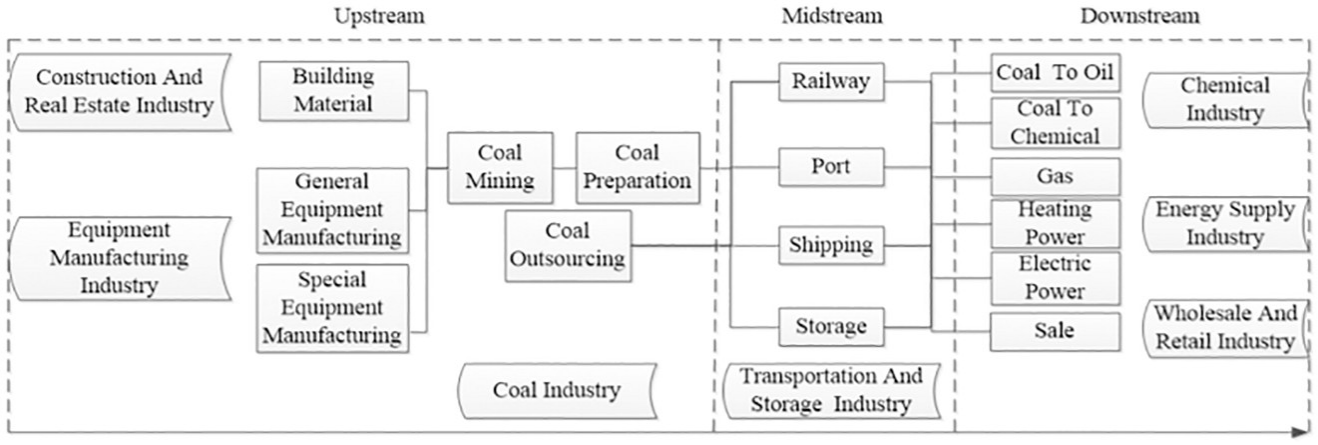
Vertical integration of coal enterprises along the industry chain.

As the promoter and practitioner of clean energy, the coal industry is also the actor and provider of clean energy technology program [5]. The ICSR of coal enterprise is a critical way to ensure that the vast reserves of coal resources in the world will not affect global warming and environmental deterioration in the use of future generations. In China, the government has recognized the importance of environmental protection [6], but enterprises are the real carriers of environmental protection policies. China’s coal enterprises are micro-carriers of fulfilling social responsibility. In recent years, the decline of coal demand, the high investment in fixed assets, the adjustment of tax structure, the continuous decrease of coal prices and the loss of the whole coal industry have seriously threatened the performance of CSR. The coal enterprises urgently need to find an appropriate strategic model to ensure the effective performance of ICSR. Starting from the goal of sustainable development, coal enterprises shoulder the historical mission of promoting the revolution of clean energy production and consumption. Through the development of “coal-gas, coal-oil, coal-electricity” and other integrated industries(Fig 2), coal enterprises focus on technological research and development, such as green coal mining, ultra-low emissions of coal-fired power plants, coal chemical industry, heavy-haul railway transportation and so on. The production chain of enterprises is gradually expanding to the whole industry chain, the efficiency of integrated operation is constantly improving, the coordination of various business sectors is further strengthened, and the industrial layout is further optimized. Through integration, clean production and consumption of coal can be realized, and the structural adjustment of coal enterprises can be fulfilled.

The existing literature has not reached a consensus on the relationship between vertical integration and ICSR, mainly because when discussing the relationship between them, it does not pay attention to the transmission way through which vertical integration affects ICSR. This paper attempts to take the financing structure and market power as intermediaries, and explore the consequences of coal enterprises’ vertical integration through chain transmission. Specifically, this paper mainly studies the following issues: (1) Can coal enterprises choose vertical integration strategy to enhance ICSR? (2) Is there a chain transmission mechanism between the change of financing structure and the promotion of market power? (3) Will this transmission mechanism be affected by the marketization process in different regions?

**Fig 3:**
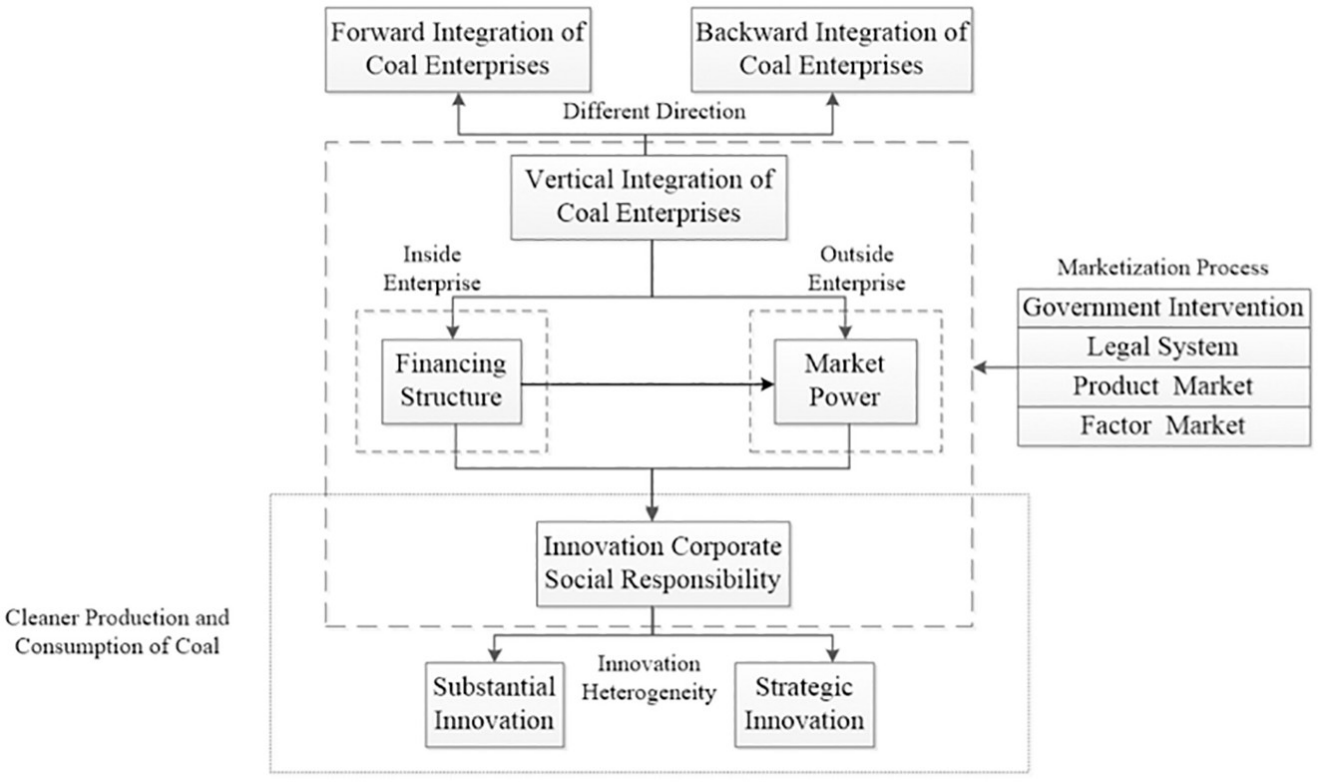
Research framework.

The answer to the above questions is the focus of the paper. Compared with the existing research, the innovation of this paper is to clarify whether the vertical integration can help to enhance the ICSR of coal enterprises, and introduce financing structure and market power as intermediary variables to study the relationship between the four factors. The organizational structure of this paper is as follows: The second part reviews the relevant literature and puts forward hypotheses. The third part lists the research methods of this paper, including variable selection, model building, and data sources. The fourth part describes the empirical results in detail and makes further research. The fifth part further discusses the empirical results. Finally, in the sixth part, we draw conclusions based on the empirical results.(Fig 3)

## Literature review and hypotheses

### The relationship between vertical integration and ICSR

In 1937, Coase [7] discussed the reasons for the existence of enterprises in his article “The Nature of Enterprises” and raised two questions. (1) What are the determinants of corporate boundaries? (2) Will the boundaries of integration affect resource allocation and corporate value? [8] Although Coase studies the nature, existence, and development reasons of enterprises in his paper, this is the theoretical source of enterprises’ industrial chain’ development. The specialized division of labor and the formation of enterprises will inevitably lead to the formation of transactions between enterprises. When the transaction cost of the external market is higher than the coordination cost of the internal departments, vertical integration can internalize the external transaction, save transaction costs, and produce economic benefits [7]. Vertical integration refers to the integration of several adjacent production stages or enterprises with input-output relationship [9]. According to the different integration directions along the industrial chain, the vertical integration of enterprises can be divided into forward integration (extending downstream of the industrial chain) and backward integration (extending upstream of the industrial chain) [10]. The driving factors and value effects of enterprises’ vertical integration include: reducing transaction costs caused by uncertainty in external markets and improve the efficiency of specialized assets [11–13]; avoiding price fluctuation of important raw materials and ensuring input of important raw materials [14]; avoiding government regulation, restrictions and taxes [15]; improving the optimal allocation of enterprise resources, thereby improving the production efficiency and corporate value of enterprises [16–18].

Enterprise performance, especially innovation performance, is the core factor affecting ICSR performance. The impact of vertical integration on corporate performance is not a new topic. As early as 1955, Frankel [19] attributed the slow development of industrial innovation in Britain from the end of the 19th century to the beginning of the 20th century to the lack of vertical integration between textile and steel industries. In 1964, Kindleberger [20] put forward the opposite view that vertical integration will inhibit the development ability of enterprises and reduce the performance of enterprises because integration will distribute the profits of enterprises to other enterprises. In empirical research, the current literature on horizontal integration of enterprise and innovation performance has been more fully studied than vertical integration, and many pieces of literature only explore the direct relationship between vertical integration and enterprise innovation performance. Because of the differences in the selected samples, the measurement of indicators and the construction of models, different conclusions have been drawn. When Banerjee and Lin [21] studied the innovation incentives of downstream firms, they found that under vertical constraints, the profits of downstream firms would increase the costs of other rivals’ downstream firms. Buehler and Schmutzler [22] found that choosing integrated enterprises under vertical structure would threaten enterprises that did not integrate, reduce the innovation performance of non-integrated enterprises while improving their own. Lahiri [23] selected 282 companies in the semiconductor industry as the research object. The research found that vertical integration helps enterprises to increase new knowledge elements, thus promoting enterprise innovation and ensuring the performance of ICSR. Karantininis et al. [24] studied 444 companies in the Danish food industry from 2000 to 2005 and found that vertical integration and contractual arrangements are important determinants of ICSR performance. Also, the integration direction and economies of scale also act a critical important role. However, Kapoor and Adner [25] find that there is a non-linear inverted U-shaped relationship between vertical integration and enterprise innovation performance. Langlois and Robertson [26] argue that either specialized centralization or vertical integration is not a magic cure for innovation performance. Enterprises should choose their business models according to the nature and scope of technological changes in the industry and the lifecycle models of various products.

In terms of empirical research, the impact of the enterprises’ vertical integration on innovation performance has not yet reached a consensus. Some scholars believe that vertical integration of enterprises has a positive impact on corporate performance (including innovation performance) [21–24]. Some scholars hold the opposite view [20]. According to transaction cost theory, industrial economics, resource-based theory, and knowledge-based theory, we consider that vertical integration of coal enterprises is beneficial to reducing transaction costs and saving enterprise costs [7, 12]. Besides, it can also avoid the fluctuation of raw material supply caused by uncertainty [14], improve the market power of enterprises [27], and increase new knowledge elements of enterprises [23] to achieve knowledge synergy effect, thereby ensuring the performance of ICSR. In order to verify the relationship between the degree of vertical integration and ICSR, we propose the first hypothesis in this paper.

Hypothesis 1(H1). Vertical integration of coal enterprises plays a direct role in promoting ICSR.

### The intermediary role of financing structure and market power

#### The intermediary role of financing structure in vertical integration and ICSR

Financing is an essential channel for enterprises to maintain normal operating cash flow, including endogenous financing, debt financing, and equity financing. The financing structure of enterprise reflects the proportion of capital sources and is an important component of internal governance. Bougheas [28] studied small and medium-sized enterprises in developed countries such as the United Kingdom, the United States and Canada. It was found that small and medium-sized enterprises tend to prefer endogenous financing because exogenous financing often faces higher financial risks. With the continuous development and improvement of China’s financial market, the proportion of exogenous financing is increasing year by year, but endogenous financing is still the primary source of enterprises’ funds. Based on China’s unique political and capital market background from other countries and regions, enterprises can accumulate unabsorbed redundancy to reduce their dependence on exogenous financing through strategic mergers and acquisitions or strategic alliances with governments, other enterprises, and organizations [29]. Dewaelheyns [30] and Twairesh [31] have shown that endogenous financing will better support the development and performance of enterprises compared with the harder-to-acquire exogenous financing. Titman and Wessels found a significant negative correlation between the proportion of loan to total assets and corporate profits. Debt financing is not conducive to corporate performance [32]. Booth, Frank and Chen [33–35] proved this point through the research of underdeveloped countries’ enterprises, American non-financial enterprises, and Chinese listed companies, respectively. Morck et al. [36] stated that when the most significant shareholder holds a large proportion of shares and the enterprise relies too much on equity financing, the daily operation of enterprises’ managers will be affected, thereby influencing the performance of enterprises. Therefore, compared with exogenous financing, endogenous financing will be more conducive to the development of enterprises. Bougheas [28] also found that although small and medium-sized enterprises in Japan, Germany, and France can finance R&D through bank loans, similar enterprises in the United Kingdom, the United States, Canada, and other regions can only rely on endogenous financing for R&D to enhance their ICSR. Goto’s research on R&D investment in the Japanese manufacturing industry specified that R&D investment of enterprises is affected by the ratio of cash flow to assets. Adequate endogenous financing is beneficial to ICSR performance [37].

In the process of vertical integration, through strategic mergers and acquisitions of upstream and downstream enterprises, enterprises have realized the substitution of internal transactions for external market transactions, saved transaction costs, formed capital integration of upstream and downstream enterprises [11–13], and completed the accumulation of unabsorbed redundancy [29]. These create conditions for enterprises to accumulate internal funds and helps enterprises to increase the proportion of endogenous financing. In the current environment, the government promotes economic development through supply-side reform and coal industry capacity removal. Compared with the coal industry, the natural gas and electricity industries in the downstream of the coal industry chain enjoy more government tax fiscal preferential policies. Therefore, coal enterprises are motivated to merge the upstream and downstream enterprises vertically and use the redundant resources of these industries to reduce the dependence of enterprises on exogenous financing, thus changing the financing structure of enterprises. The increase of endogenous financing can improve the performance of enterprise [30, 31], thereby ensuring the performance of ICSR in coal enterprises. At the same time, compared with exogenous financing, R&D innovation activities of enterprises are more dependent on endogenous financing [28, 37], which is stronger operability, less external dependence and fewer use conditions. Accordingly, the second hypothesis of this paper can be put forward.

Hypothesis 2(H2). The change of financing structure plays an intermediary role in the incentive of vertical integration to ICSR of coal enterprises.

#### The intermediary role of market power in vertical integration and ICSR

Market power is an important index to measure the position of enterprises in the industry. It is generally defined as the ability of enterprises to raise prices above marginal costs [38]. Theorists generally believe that market power is one of the main motivations for the vertical integration of enterprises [39, 40]. Stigler [15] believes that enterprises internalize external economy through vertical integration, which can correct market failure caused by externalization, enhance the market power of enterprises, or reduce the impact of market power on enterprises, thus realizing price discrimination. As early as 1967, Comanor pointed out in his book that enterprises control the key resources of upstream and downstream enterprises in the industrial chain through vertical integration mergers and acquisitions, and avoid other enterprises entering the industry, thus indirectly improving their market power and forming monopolistic industrial behavior. Williamson also believes that mergers and acquisitions are sometimes not to enhance the efficiency of enterprises, but to pursue or enhance their market power, achieve industry monopoly, and then obtain monopoly profits. With the improvement of the market power of enterprises, the ability of enterprises to control the price of products has been strengthened. Enterprises have a monopoly position in the industry, which increases their confidence in devoting themselves to unstable R&D and innovation activities. Schumpeter’s innovation theory put forward in 1942 holds that a certain degree of market power creates conditions for enterprise’s technological innovation and brings innovation power, thus promoting technological innovation and guaranteeing the enterprise’s ICSR performance. Blundell et al. found that although fierce competition in the industry is beneficial to stimulate innovation activities, higher market power can often have a positive effect on the observed innovation behavior and patent number of enterprises. Enterprises with higher market power can often have incentives to pre-emptively innovate [41].

Generally speaking, market power theory includes two mechanisms: structuralism mechanism and behavioral mechanism. However, no matter what kind of mechanism, the reason for the vertical integration of enterprises along the industrial chain is to obtain market power [15, 39, 40], and the size of market power is regarded as the decisive factor for the ability of enterprises to control the price of products [38]. Therefore, the promotion of market power can effectively improve the economic performance [42] and R&D investment [41] of enterprises. At the same time, vertical integration of coal enterprises is in line with the trend of supply-side reform in China’s coal industry, so it is more conducive to obtaining government R&D support. According to industrial economics, vertical integration can effectively enhance the market power of enterprises [15, 27, 39, 40]. Enterprises have control over the price of products [38], which improves the economic performance of them [42]. According to the innovation theory, it provides the motive force for the innovation activities of enterprises, thus guaranteeing the performance of ICSR. Accordingly, the third hypothesis of this paper can be put forward.

Hypothesis 3(H3). The improvement of market power plays an intermediary role in the incentive of vertical integration to ICSR of coal enterprises.

#### The chain intermediary role of financing structure and market power in vertical integration and ICSR

Through the above analysis, it can be found that the vertical integration of coal enterprises can ensure ICSR not only by changing the financing structure of enterprises but also by improving market power. So what is the relationship between financing structure and market power in the promoting of enterprises’ vertical integration to ICSR, and whether there is a chain transmission mechanism between them? The paper holds that financing structure is the internal factor affecting ICSR, and market power is the external factor affecting ICSR. Vertical integration of coal enterprises through the internalization of the external market, realizes the savings of transaction costs [7, 12], completes the unified allocation of capital of upstream and downstream enterprises [16–18] and accumulates unabsorbed redundancy within the organization [29], thus reducing the dependence of enterprises on exogenous financing, and changes the financing structure of enterprises. The change of financing structure enables enterprises to use their internal funds more freely, seize the changing market opportunities and achieve higher market power. The promotion of market power will not only enrich the resources of enterprises [38] but also increase the confidence of enterprises in R&D innovation [41]. This will ensure the performance of ICSR. Based on the above analysis, this paper continues to put forward the following research hypotheses:

Hypothesis 4(H4). The change of financing structure and the promotion of market power play a chain intermediary role in the incentive of vertical integration to ICSR of coal enterprises.

### The impact of marketization process on vertical integration and ICSR

The market environment is an important factor affecting enterprise behavior [43]. In areas with high marketization process, the government has less market intervention. Relatively, these areas have a high level of the legal system, and the development of product and factor markets is perfect [44]. Enterprises can realize the unified allocation and synergy effect of resources by vertical integration through sound market mechanism according to their own needs, obtain lasting competitive advantages, and then ensure the performance of ICSR [16–18]. Wu’s research suggests that the improvement of the market environment is beneficial to the improvement of technological innovation level [45]. However, in the areas with low marketization process, government intervention is high. The legal system in these areas is imperfect and the development of product and factor markets is incomplete [44–46]. Therefore, enterprises need to spend a lot of time and resources to cope with the mismatch of government functions. Coupled with the inadequate protection of intellectual property rights and the inability to obtain innovative resources at a lower cost, the intention of coal enterprises to implement ICSR is often low. On this basis, the paper puts forward further hypotheses:

Hypothesis 5(H5). In areas with higher marketization process, vertical integration has more significant incentive effect on ICSR.

**Fig 4:**
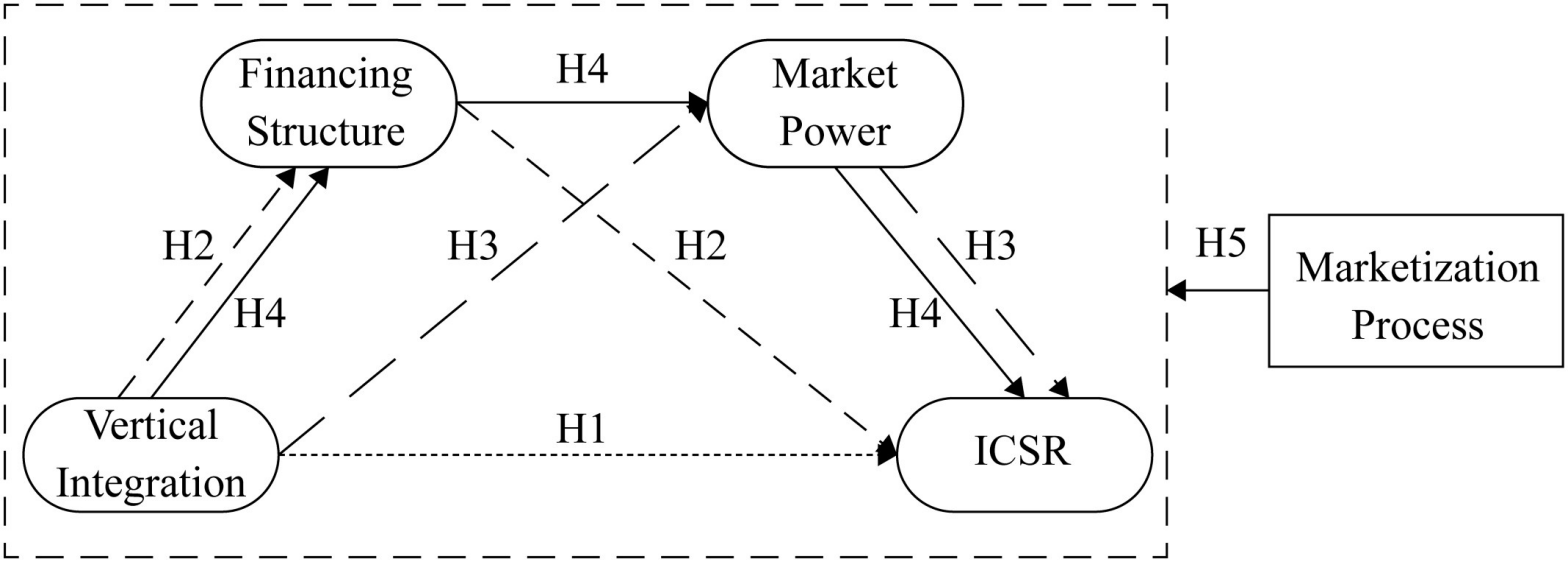
Conceptual model.

The research hypothesis of this paper is as follows(Fig 4):

## Empirical design

### Selection of variables

Vertical integration of enterprises can be divided into forward integration and backward integration. In this paper, *Forward* is used to express the degree of forward integration of coal enterprises; *Backward* is used to express the degree of backward integration of coal enterprises. At the same time, *FS* is used to represent the financing structure of enterprises, *MP* is used to represent the market power, and Patent is used to represent ICSR performance. The specific calculation method is shown in Table 1.

**Table 1:** Indicators of different study variables.

Study variables of coal enterprises	Proxies	Definition
Forward integration	Forward	Industry matching
		Calculating the input-output index $\vartheta_{i}=\sum_{j=1,j\neq i}^{n}\vartheta_{ij}$
		Weighted summation $V_{forw}=\sum_{i=1}^{n}w_{i}\vartheta_{i}$
Backward integration	Backward	Industry matching
		Calculating the input-output index $\theta_{i}=\sum_{j=1,j\neq i}^{n}\theta_{ji}$
		Weighted summation $V_{backw}=\sum_{i=1}^{n}w_{i}\theta_{i}$
Financing structure	FS	FS = Short term loan/Total assets
Market power	MP	MP = Revenue/Total revenue of mining industry
ICSR	Patent	Patent = ln(Patent authorizations+1)

#### Explained variable

ICSR refers to the spontaneous awareness of the impact of technological innovation on sustainable development and business success. Enterprises promote technological progress through innovation, thus consciously assuming the responsibility of economic and social development and environmental protection. As for the measurement of ICSR performance, the current literature commonly uses enterprises’ R&D input-output ratio, patents authorization, the revenue of new products, the proportion of intangible assets to total assets [47] to measure. Considering the persistent impairment of intangible assets and too many subjects under it, the revenue of new products reflecting the industrialization performance of innovative achievements, and the uncertainty of R&D investment and the long cycle, this paper chooses the number of patents authorization of enterprise in this year as the measurement index of ICSR. Because of the dimension difference between patents authorization and other variables expressed by percentages, such as vertical integration, financing structure and market power, we standardize it to eliminate the impact of quantity, and scale to make the results more intuitive.

#### Explanatory variables

Vertical integration refers to the joint operation of enterprises with users or suppliers, or the direct extension of the business to upstream and downstream industries. It can form the integrated operation mode of supply-production, production-marketing or supply-production-marketing, so as to expand the scope of business. This paper focuses on the vertical integration model that enterprises directly extend their business to upstream and downstream industries. Current literature about the measurement of the degree of vertical integration can be summarized into four types: value-added method [48], primary and secondary classification method [49], input-output table method [50], and Davies-Morris index method [51]. Considering the validity of data and the universality of the input-output table method, this paper combines the input-output table method and financial data (divisional operating revenue) to measure the degree of vertical integration. Before calculating, it is necessary to match the industries involved in the main business disclosed in the annual report of an enterprise with those in the input-output table (hereinafter referred to as the I-O table).

Firstly, the degree of forward integration is calculated. The specific calculation steps are as follows:

1. Calculate the input-output index. According to the basic flow table in the I-O table, we calculate the outflow of per unit output of industry “*i*” to other industry “*j*” (where “*i*” and “*j*” are the industries where the branches of enterprises are located). Then we sum the coefficient to get the total outflow of per unit output of industry “*i*” to other industries.
  $$\begin{array}{c} \vartheta_{i}=\sum_{j=1,j\neq i}^{n}\vartheta_{ij} \end{array}$$ (1)
2. Weighted summation. This step involves calculating the proportion of the industry revenue involved in the enterprises against the total revenue of the enterprises. The proportion as weight is taken, and the input-output index is summed up. Finally, the degree of forward integration of enterprises is arrived upon.
  $$\begin{array}{c} V_{forw}=\sum_{i=1}^{n}w_{i}\vartheta_{i} \end{array}$$ (2)
  *w*_*i*_ represents the divisional revenue of the industry “*i*” accounting for the proportion of total revenue.

The calculation of the degree of backward integration is similar to that of forward integration. The specific steps are as follows:

1. Calculate the input-output index. According to the basic flow table in the I-O table, we calculate the inflow, which per unit output of industry “*i*” needs, from other industry “*j*” (“*i*” and “*j*” are the industries where the branches of the enterprise are located). Then we sum the coefficient to get the total inflow from the other industries involved in the per unit output of industry “*i*”.
  $$\begin{array}{c} \theta_{i}=\sum_{j=1,j\neq i}^{n}\theta_{ji} \end{array}$$ (3)
2. Weighted summation. This step involves calculating the proportion of the industry revenue involved in the enterprises against the total revenue of the enterprises. The proportion as weight is taken, and the input-output index is summed up. Finally, the degree of backward integration of enterprises is arrived upon.
  $$\begin{array}{c} V_{backw}=\sum_{i=1}^{n}w_{i}\theta_{i} \end{array}$$ (4)
  The domain of the index is restricted to the interval 0 ≤ *w*_*i*_ < 1. The bigger this index is, the greater the extent of the vertical integration will be.

#### Mediate variables

In this paper, the financial structure index (*FS*) is used to reflect the dependence of enterprises on exogenous debt funds. Considering that the main source of debt funds for Chinese enterprises is financial institutions such as banks, the proportion of short-term loans to total assets at the end of the year is used to calculate it. The market power index (*MP*) is used to reflect the enterprises’ market share. The Common measures of market power include the plus rate and Lerner index [52]. Considering the significant impact of China’s coal industry on the whole mining industry, this paper uses the proportion of the coal enterprise’s revenue to total revenue of the mining industry to calculate it.

#### Control variables

The paper mainly includes the following control variables. (1) ICR represents corporate financial leverage, which is the index to measure the solvency of an enterprise. It equals the net cash flow from business operation divided by liabilities. (2) Invint denotes inventory intensity, which is an indicator of enterprise’s capital structure. It equals the total inventory divided by the total assets at the end of the year. The higher the inventory intensity, the lower the proportion of fixed assets. (3) Sholder is the proportion of the enterprise’s payment held by major shareholders, which is the ratio of other receivables to total assets at the end of the year.

### Model design

In order to test the direct incentive effect of vertical integration on ICSR, this paper constructs a multiple linear regression model to verify it.

$$\begin{array}{c} \begin{array}{cc} Patent & =\beta_{0}+\beta_{1}\times VI+\beta_{2}\times ICR+\beta_{3}\times Invint+\beta_{4}\times Sholder\\ & +\sum \beta_{j}\times Year+\eta \end{array} \end{array}$$

*Patent* represents the ICSR performance, *VI* represents the vertical integration degree of the enterprise, and *η* is the residual term. According to the extension direction of the industry chain, vertical integration(*VI*) of enterprises can be divided into forward integration and backward integration, in which forward integration is expressed by *Forward* and backward integration by *Backward*. In addition, three control variables are used to control the changes of other factors. *ICR* represents corporate financial leverage, *Invint* denotes inventory intensity, and *Sholder* is the proportion of enterprise’s payment held by major shareholders.

In the research of sociology and psychology, after exploring whether there is a correlation between the two factors and how significant the correlation is, we will want to further research why this relationship exists, and then realize the so-called mechanism research by mining intermediary variables. From both theoretical and practical perspectives, the complex relationship between the two factors cannot be explained by a single mediating variable. When multiple mediating variables exist, they inevitably interact with each other, forming a complex relationship with the antecedents and results. In order to clearly depict these complex relationships, Multi-Step Multiple Mediator Model emerged as the times require. Considering the defect of causal step regression [53] in the validity of the test method and the rationality of the test procedure [54], this paper uses the Bootstrap method proposed by Preacher and Hayes [54] to test the chain mediation effect between financing structure and market power. Preacher and Hayes [54] believe that the test of intermediary effect should be used to determine whether the intermediary path exists or not, which means there is no need to test the main effect, that is, whether the vertical integration of coal enterprises promotes ICSR or not. However, in order to ensure the integrity of the paper, the paper still uses multiple linear regression method to verify it. This paper uses the Bootstrap method to test the mediation and chain mediation effect of financing structure and market power. Because the Bootstrap method is often used to test the existence of mediation relationship directly, there is no need to add control variables in the test, so this paper does not build models for Hypothesis 2-4. Instead, the Bootstrap method is used to sample 5000 times. Moreover, 95% unbiased correction confidence interval is constructed to observe whether the confidence interval of indirect effect includes 0. If not, the mediating effect is significant. If the confidence interval of direct effect includes 0, the mediating variable is the only mediating variable; if the confidence interval of direct effect does not include 0, there are still other mediating variables.

### Data source and description

#### Data sources

The samples selected are coal enterprises, which are Chinese listed companies in the Shenzhen Stock Exchange and Shanghai Stock Exchange from 2008 to 2017. The sample firms’ main business centers on coal mining and coal washing. These enterprises are also experienced at vertical integration. Of these companies, the present paper first eliminates special-treated firms (firms that have suffered losses for two consecutive fiscal years), marked as “ST” and “*ST”. Companies with missing or abnormal financial data were then excluded. As a result, the paper compiled a sample of 200 coal enterprises in China from 2008 to 2017.

Annual Report data are available from the Shanghai Stock Exchange (www.sse.com.cn), Sina Finance’s website (finance.sina.com.cn), and the Shenzhen Stock Exchange (www.szse.cn). Other financial data are available from the CSMAR (www.gtarsc.com) and CCER (www.ccerdata.cn) databases. The CSMAR and CCER databases are commercially available from the Chinese Academy of Science. Data for vertical integration are based on table I-O of 2012, released on the website of the National Bureau of Statistics of the People’s Republic of China (www.stats.gov.cn), and annual reports released by listed companies. The statistical software used in this paper includes SPSS21.0 and Stata 14.

#### Description

Table 2 shows the main descriptive statistics for each variable. The maximum value of ICSR (*Patent*) is 6.9017 and the minimum value is 0, which indicates that the ICSR performance in the coal industry is quite different. In addition, the mean value is 1.8237, which indicates that most enterprises can still maintain the basic performance of ICSR. The average value of forward integration (*Forward*) is 18.09%, the minimum value is 0, and the maximum value is 49.17%. This shows that the forward integration degree of Chinese coal enterprises is different. The average value of backward integration (*Backward*) is 4.37%, the minimum value is 0, and the maximum value is 14.99%. This shows that compared with forward integration, the degree of backward integration of China’s coal enterprises is lower, which is closely related to the position of the coal industry in the upstream of the industrial chain. The difference between financing structure (*FS*) and market power (*MP*) among enterprises is also large, which is consistent with the current situation of the development of China’s coal enterprises. The average value of each index is close to the median, which shows that the indexes of coal enterprises basically conform to the normal distribution and the current situation of China’s coal enterprises. From the analysis above, it is clear that the sample selection is objective.

**Table 2:** Descriptive statistics on listed Chinese coal companies.

	N	Mean	P25	Median	P75	SD	Var	Min	Max
Patent	200	1.8237	0.0000	1.6094	3.1781	1.8088	3.2717	0.0000	6.9017
Forward	200	0.1809	0.0375	0.1078	0.3747	0.1646	0.0271	0.0000	0.4917
Backward	200	0.0437	0.0197	0.0405	0.0711	0.0324	0.0010	0.0000	0.1499
FS	200	0.0954	0.0262	0.0565	0.1423	0.0908	0.0082	0.0000	0.3993
MP	200	0.0055	0.0012	0.0028	0.0051	0.0086	0.0001	0.0003	0.0458
ICR	200	0.2952	0.0701	0.1955	0.4488	0.3282	0.1077	-0.2873	1.4462
Invint	200	0.0445	0.0181	0.0278	0.0555	0.0494	0.0024	0.0036	0.4098
Sholder	200	0.0157	0.0043	0.0089	0.0183	0.0230	0.0005	0.0002	0.2294

## Empirical results and analysis

### Hypothesis testing

#### Vertical integration and ICSR

This paper uses a linear regression model to test the impact of vertical integration on ICSR of coal enterprises and the empirical results are as shown in Table 3. The explained variable is ICSR (*Patent*), and the explanatory variables are forward integration (*Forward*) and backward integration (*Backward*). As can be seen from the empirical results, the regression coefficient between forward integration degree and ICSR is 1.6192, and there is a significant positive correlation at 5% level (see Table 3, Line 3, Column 2). Meanwhile, the regression coefficient between backward integration degree and ICSR is 18.2552, and there is a significant positive correlation at 1% level (see Table 3, Line 4, Column 4). This shows that the vertical integration strategy helps to make full use of the advantages, promote capital and technology integration of upstream and downstream enterprises, and then improve the performance of ICSR. For every 1% increase in the degree of forward integration of coal enterprises, the ICSR performance will correspondingly increase by 1.62%, and for every 1% increase in the degree of backward integration, the ICSR performance will correspondingly increase by 18.26%. This verifies Hypothesis 1 that vertical integration has a positive effect on ICSR performance of coal enterprises, and backward integration seems to have a more significant impact on the performance of ICSR. According to the regression coefficient of the other variables in Table 3, there is a significant positive correlation between corporate financial leverage (*ICR*) and ICSR performance. Enterprises have higher solvency, which means that enterprises have more liquidity and managers have more funds at their disposal. So, more funds can be used for R&D. Stable R&D investment has brought about the improvement of innovation ability and ensured the performance of ICSR.

**Table 3:** Vertical integration and ICSR regression results.

	Estimated coefficients	T value	Estimated coefficients	T value
Intercept	-0.7728	-1.27	-0.7268	-1.29
Forward	1.6192**	2.08		
Backward			18.2552***	4.84
ICR	1.8005***	3.64	1.3836***	2.87
Invint	2.3574	0.82	-1.3068	-0.48
Sholder	3.2811	0.59	6.4303	1.22
Year effect	Controlled		Controlled	
AdjR2	0.1266		0.2063	
F-statistic	3.22***		4.98***	
N value	200		200	

Notes:

“***”, “**”, and “*” represent the significance level of 1%, 5% and 10% respectively.

#### Intermediary test of financing structure

In order to test the intermediary role of financing structure(*FS*) in the incentive of vertical integration to ICSR performance of coal enterprises, the Bootstrap method is used to sample 5000 times and build 95% unbiased correction confidence interval. The results show that the intermediary effect of forward integration on ICSR is 0.5343, and the 95% confidence interval is [0.0019, 1.2117] (see Table 4, Line 4, Column 2-4). It excludes 0, indicating that the intermediary effect of financing structure is significant. The intermediary effect of backward integration on ICSR is 1.3183, and the 95% confidence interval is [0.1383, 3.3248] (see Table 4, Line 5, Column 2-4). It excludes 0, also indicating that the intermediary effect of financing structure is significant. At the same time, both forward integration and backward integration have significant direct effects on ICSR, which indicates that financing structure plays a partial intermediary role in the incentive of vertical integration to ICSR. Therefore, Hypothesis 2 has been proved. The test results are as shown in Table 4.

**Table 4:** Intermediary test of financing structure.

Ind	Indirect effect			Direct effect		
	Effect	95%confidence interval		Effect	95%confidence interval	
		BootLLCI	BootULCI		LLCI	ULCI
Forward→FS→Patent	0.5343	0.0019	1.2117	1.6704	0.0435	3.2973
Backward→FS→Patent	1.3183	0.1383	3.3248	19.3649	11.9519	26.7779

#### Intermediary test of market power

This paper continues to use the Bootstrap method to test the intermediary role of market power (*MP*) in the incentive of vertical integration to ICSR of coal enterprises. The results show that in the influence of forward integration on ICSR, the intermediary effect of market power is 0.9432, and the 95% confidence interval is [0.3096, 1.7515] (see Table 5, Line 4, Column 2-4). It excludes 0, which indicates that the intermediary effect of market power is significant. In the influence of backward integration on ICSR, the intermediary effect of market power is 11.4906, and the 95% confidence interval is [5.1682, 18.6484] (see Table 5, Line 5, Column 2-4). It excludes 0, which also shows that market power has significant intermediary effects. The direct effect of forward and backward integration on ICSR is significant, which indicates that market power plays a partial intermediary role in the incentive of vertical integration to ICSR. Therefore, Hypothesis 3 has been proved. The test results are as shown in Table 5.

**Table 5:** Intermediary test of market power.

Ind	Indirect effect			Direct effect		
	Effect	95%confidence interval		Effect	95%confidence interval	
		BootLLCI	BootULCI		LLCI	ULCI
Forward→MP→Patent	0.9432	0.3096	1.7515	1.2616	0.0261	2.4970
Backward→MP→Patent	11.4906	5.1682	18.6484	9.1926	2.5216	15.8636

#### Chain intermediary test of financing structure and market power

**Fig 5:**
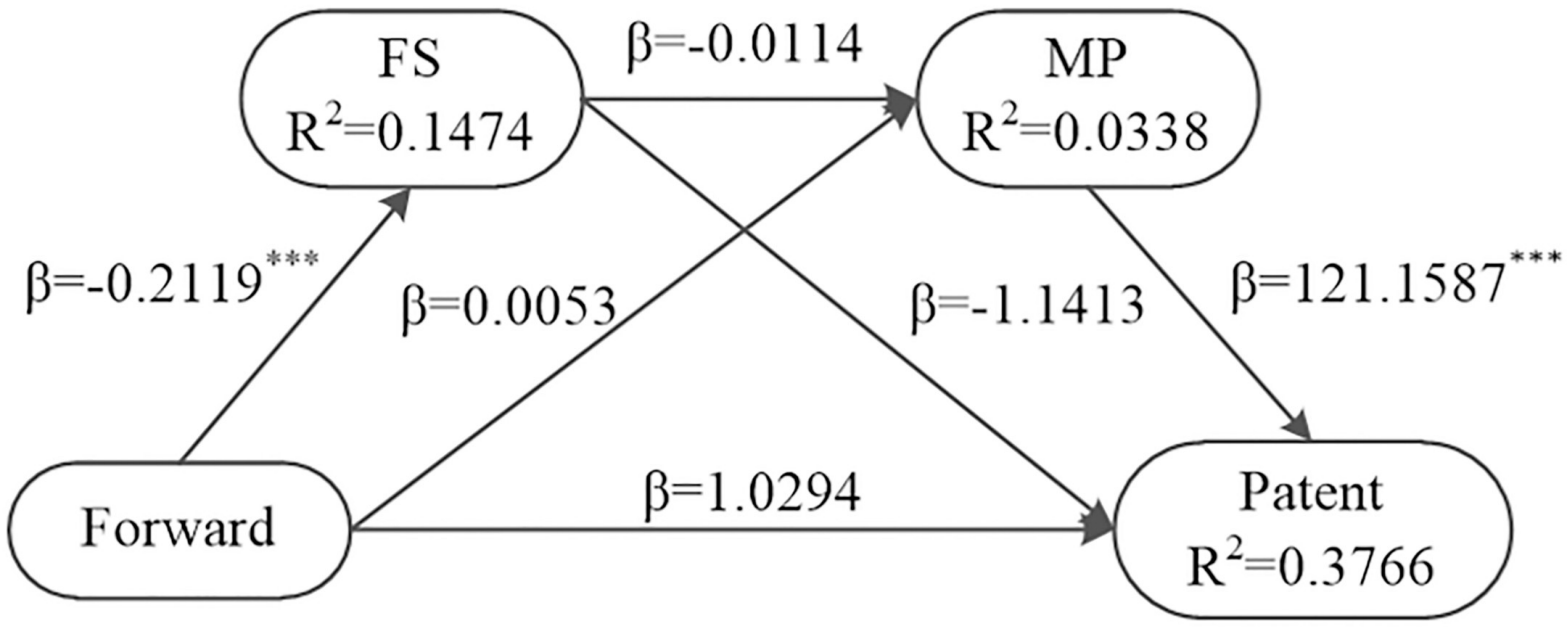
Chain intermediary test of financing structure and market power with forward integration.

The paper still uses the Bootstrap method to test the chain intermediary role of financing structure and market power in the incentive of forward integration to ICSR of coal enterprises. The results show that the chain intermediary effect of financing structure and market power is 0.2925, and the 95% confidence interval is [0.0350, 0.6207], excluding 0 (see Table 6, Line 6, Column 2-4). This shows that the chain intermediary effect of financing structure and market power is significant. Meanwhile, the direct effect of forward integration on ICSR is 1.0294, and the 95% confidence interval is [-0.3006, 2.3593], including 0 (see Table 6, Line 4, Column 5-7). This indicates that the chain intermediary plays a full intermediary role. The test results are as shown in Table 6, Fig 5.

**Table 6:** Chain intermediary test of financing structure and market power with forward integration.

Ind	Indirect effect			Direct effect		
	Effect	95%confidence interval		Effect	95%confidence interval	
		BootLLCI	BootULCI		LLCI	ULCI
Total	1.1754	0.4040	2.0745	1.0294	-0.3006	2.3593
Forward→FS→Patent	0.2419	-0.1991	0.7541			
Forward→FS→MP→Patent	0.2925	0.0350	0.6207			
Forward→MP→Patent	0.6411	0.0462	1.3633			

**Fig 6:**
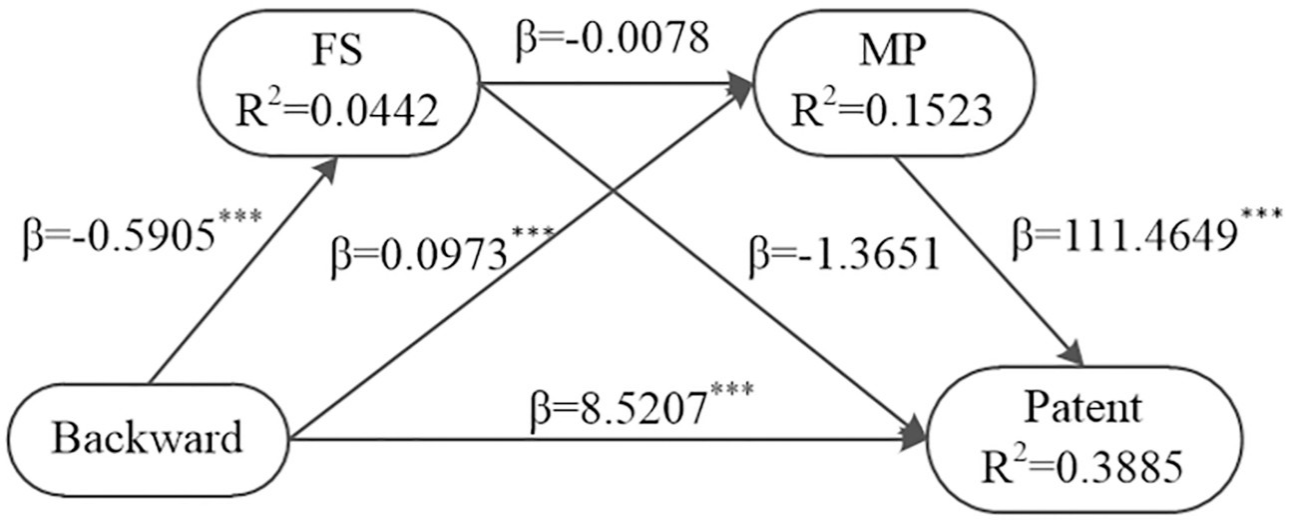
Chain intermediary test of financing structure and market power with backward integration.

Bootstrap test on the chain intermediary role of financing structure and market power in the incentive of backward integration to ICSR of coal enterprises shows that the chain intermediary effect is 0.5122, and the 95% confidence interval is [0.0167, 1.2180], excluding 0(see Table 7, Line 6, Column 2-4). It shows that the chain intermediary effect of financing structure and market power is significant. In addition, the direct effect of backward integration on ICSR is 8.5207, the 95% confidence interval is [1.7652, 15.2762](see Table 7, Line 4, Column 5-7). It does not contain 0, indicating that the chain intermediary plays a partial intermediary role. Therefore, Hypothesis 4 has been proved. The test results are as shown in Table 7, Fig 6.

**Table 7:** Chain intermediary test of financing structure and market power with backward integration.

Ind	Indirect effect			Direct effect		
	Effect	95%confidence interval		Effect	95%confidence interval	
		BootLLCI	BootULCI		LLCI	ULCI
Total	12.1625	5.9712	19.1604	8.5207	1.7652	15.2762
Backward→FS→Patent	0.8061	-0.2310	2.4287			
Backward→FS→MP→Patent	0.5122	0.0167	1.2180			
Backward→MP→Patent	10.8442	4.9038	17.5349			

### Further research based on heterogeneous of marketization process

Referring to Fan Gang’s “**China’s Provincial Marketization Index Report 2016**” [44], this paper ranks the total index of the provincial marketization process from high to low. The latter 50% of the provinces are defined as the low marketization process group with a poor market environment, while the other provinces are defined as the high marketization process group with a better market environment. Based on this, the samples are divided into two groups for Bootstrap test, and the results are shown in Tables 8 and 9. As can be seen from the previous chapters and Tables 8 and 9, vertical integration has a significant impact on ICSR and the financing structure and market power play a chain intermediary role in it. This relationship is significant only in areas with higher marketization process and a better market environment, while it is almost not statistically significant in areas with lower marketization process and a poorer market environment. This proves Hypothesis 5, that is, vertical integration of coal enterprises is more significant to promote ICSR in areas with higher marketization process.

**Table 8:** Enterprises with high marketization process.

Ind	Indirect effect			Direct effect		
	Effect	95%confidence interval		Effect	95%confidence interval	
		BootLLCI	BootULCI		LLCI	ULCI
Forward→FS→Patent	2.8488	1.6078	4.4966	1.1709	-1.5847	3.9264
Backward→FS→Patent	8.4777	4.1251	14.7909	18.0455	5.7547	30.3363
Forward→MP→Patent	1.1053	0.0283	2.6882	2.9144	0.9535	4.8753
Backward→MP→Patent	19.2323	7.5200	30.8556	7.2909	-4.2725	18.8542
Forward→FS→MP→Patent	1.2246	0.5746	2.2297	1.4282	-0.6019	3.4584
Backward→FS→MP→Patent	2.4459	1.2553	4.3801	3.9099	-6.6050	14.4248

**Table 9:** Enterprises with low marketization process.

Ind	Indirect effect			Direct effect		
	Effect	95%confidence interval		Effect	95%confidence interval	
		BootLLCI	BootULCI		LLCI	ULCI
Forward→FS→Patent	-0.5701	-1.1687	-0.0829	0.2446	-1.2826	1.7717
Backward→FS→Patent	-1.5269	-4.4577	-0.1028	4.3717	-3.7959	12.5392
Forward→MP→Patent	-0.0038	-0.2084	0.0974	-0.3218	-1.7802	1.1367
Backward→MP→Patent	0.0602	-0.4840	1.4670	2.7846	-5.4582	11.0275
Forward→FS→MP→Patent	0.1174	-0.0411	0.4263	0.3773	-1.1652	1.9198
Backward→FS→MP→Patent	0.2483	-0.0454	1.4946	4.3672	-3.7950	12.5293

### Robustness test

In order to examine the robustness of the results, this paper uses the number of patent applications in the year of the enterprise instead of the number of patent authorizations as the measurement variable of ICSR. This paper examines the direct impact of coal enterprises’ vertical integration on ICSR, intermediary and chain intermediary effect, and further tests based on heterogeneity. The test results are basically consistent with the previous results, and the empirical analysis results in this paper are valid. Considering the length of the paper, only the test results of intermediary and chain intermediary effect are presented, as shown in Table 10.

**Table 10:** Robustness test.

Ind	Indirect effect			Direct effect		
	Effect	95%confidence interval		Effect	95%confidence interval	
		BootLLCI	BootULCI		LLCI	ULCI
Forward→FS→Patent	0.5857	0.0273	1.3177	2.0344	0.3644	3.7044
Backward→FS→Patent	1.4971	0.1419	3.5886	21.9393	14.4062	29.4723
Forward→MP→Patent	0.9754	0.3376	1.7857	1.6447	0.3796	2.9097
Backward→MP→Patent	11.6474	5.5246	18.4935	11.7889	4.9867	18.5912
Forward→FS→MP→Patent	0.3020	0.0490	0.6353	1.3723	0.0115	2.7332
Backward→FS→MP→Patent	0.5180	0.0087	1.2147	10.9729	4.0950	17.8508

## Discussion

First, Frankel et al [19] demonstrated a significant positive correlation between firm vertical integration and enterprise performance, but which contradicted Kindleberger et al. [20]. This result may be attributed to the dual externalities of vertical integration, that is, vertical integration exists the distribution of operating profit among different branches while promoting enterprise resource synergy. On the one hand, enterprises adopt and absorb the tangible and intangible assets of the acquired party through mergers and acquisitions. In addition, it can form a synergistic effect with the original resources of the enterprise itself, which reduces the negative externalities, strengthens the control of the market and improves the level of innovation factor reserve. Through vertical integration, coal enterprises will leave the transactions between the raw coal, natural gas and power industries upstream and downstream of the coal industry chain in the enterprise. It will save the negotiation and transaction costs between the upstream and downstream manufacturers, and form the integration of capital and technology between different industries [11], which promotes the improvement of innovation capability of coal industry enterprises and ensures the ICSR performance. On the other hand, the vertical extension of the enterprise in the industrial chain will inevitably involve the redistribution of operating profit among the branches [20]. Besides, as the scale of the enterprise expands, the management cost and administrative levels will further increase. The mastery of the innovation elements will be weakened, thus inhibiting the innovation ability of enterprises and affecting the ICSR performance. At the same time, considering that the government’s guidance is the main cause of coal enterprises’ vertical integration and fulfilling social responsibilities, coal enterprises have strategic innovations that cope with government assessment indicators to defraud rewards. Therefore, the actual ICSR performance of enterprises may not be improved.

Second, the financing structure and market power play a chain intermediary role in the incentive effect of vertical integration to ICSR performance of coal enterprises. Because the vertical integration of coal enterprises will no doubt save transaction costs and accumulate more funds through the internalization of the external market, whether through forward integration to enhance the grasp of the market, or through backward integration to maintain the stability of raw material supply. Vertical integration can reduce their dependence on exogenous financing, especially debt financing, to change the financing structure. The change in the financing structure makes the use of funds more comfortable, and the stronger the grasp of the ever-changing market opportunities, the higher the market power. At the same time, vertical integration along the industrial chain enables companies to control the critical resources of upstream and downstream, reduces the possibility of other companies entering the coal industry, and thus directly achieving a monopoly on the industry. The increase of endogenous financing and the improvement of market power make the company more attractive to high-level innovation, marketing, and management talents, increase the ability to undertake R&D risks, and then effectively allocate innovation elements to ensure the ICSR performance.

## Conclusion

China is a large resource country, and the coal industry is the main resource supplier of China’s energy economy system. Therefore, the integration effect of the coal industry chain is of great significance for improving the efficiency of resource utilization, promoting resource intensive and centralized management, ensuring clean production of coal resources, and promoting the healthy and sustainable development of the resource industry. With the acceleration of China’s supply-side structural reform and the improvement of related technology, vertical integration of coal enterprises is becoming more and more common. As to whether vertical integration can help promote ICSR performance, the existing research literature has not reached a unified conclusion. The reason is that the existing research only focuses on the direct relationship between vertical integration and ICSR performance, but neglects the way that vertical integration affects it.

### Conclusions and policy recommendations

**Fig 7:**
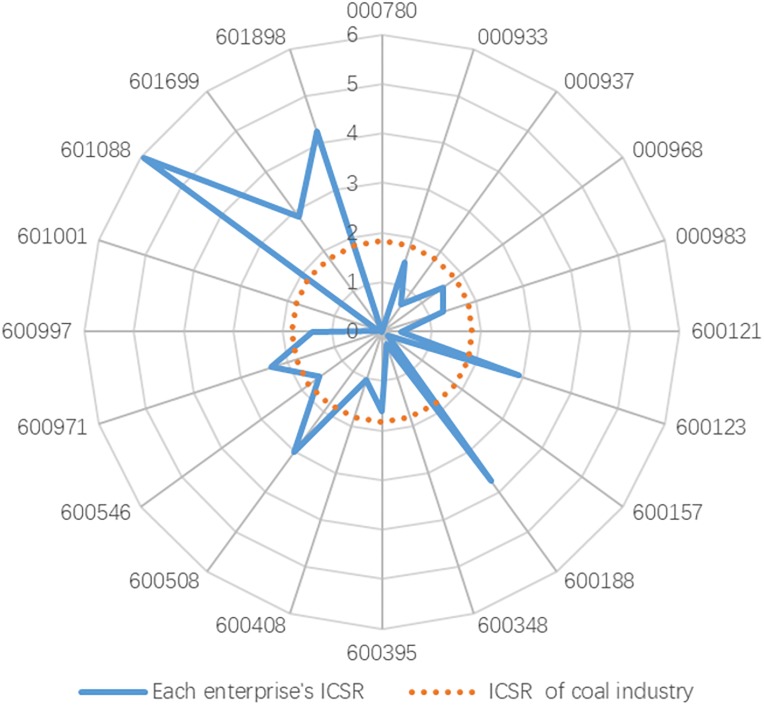
Each enterprise’s average ICSR of the Chinese coal industry, 2008-2017.

Taking 200 coal A-share listed companies of the Shenzhen Stock Exchange and Shanghai Stock Exchange in China from 2008 to 2017 as samples, this paper starts from the perspective of financing structure and market power and calculates the degree of vertical integration by input-output table method. Through multiple linear regression and Bootstrap, this paper empirically studies the chain relationship among the degree of vertical integration, financing structure, market power, and ICSR performance. The main conclusions and policy recommendations are as follows:

Firstly, for the purpose of saving costs, dispersing risks and completing the early accumulation of innovative elements, coal enterprises are motivated to unite upstream and downstream enterprises by implementing vertical integration strategy, which is manifested in a significant positive correlation between the degree of vertical integration and ICSR performance of coal enterprises. As shown in Fig 7, from 2008 to 2017, the average value of ICSR performance in the coal industry is 1.8237, and the average value of ICSR performance in 13 enterprises is lower than the average value of the whole coal industry. Under the existing technology and resources conditions, the performance of ICSR in the Chinese coal industry still has considerable room for improvement. Wu et al. [55] found that the growth of innovation performance is related to the increase of green CSR. Therefore, at this stage, China should actively promote the vertical integration of coal enterprises, realize the synergistic effect of resources, clarify the economic and social obligations of the enterprises, promote ICSR performance, and make coal enterprises become the leader of clean production of resources and ecological environmental protection.

Secondly, the savings of intermediate costs such as transaction costs and negotiation costs endow enterprises with more internal liquidity, reduce their dependence on exogenous financing, especially debt financing. It also increases the flexibility of capital use, enables enterprises to grasp fleeting market opportunities, and increases market control. The reduction of operation and financing risk and government’s R&D support enable enterprises to better engage in R&D innovation activities with higher risks. At the same time, enterprises with sufficient funds and higher market power are more likely to attract excellent management and R&D personnel and enhance the success rate of enterprise innovation output and commodity promotion. Specifically, financing structure and market power play a chain intermediary role in the promotion of ICSR by vertical integration of coal enterprises.

Thirdly, in areas with higher marketization process, enterprises can make use of a sound market mechanism to achieve the unified allocation and synergy of resources through vertical integration, to enhance innovation ability and obtain lasting competitive advantages. That is, in areas with higher marketization process, vertical integration has more significant incentive effect on ICSR from the perspective of market environment heterogeneity. Wang et al. [56] showed that the influence of institutional factors on CSR efficiency is complex and nonlinear. China government should accelerate the construction of the market, reduce the interference of local government, promote the development of product market and factor market, perfect the environment of the legal system, and provide a sound protection mechanism for innovation of coal enterprises to maintain the incentive effect of vertical integration on ICSR.

### Research contributions

Through the intermediary variables of financing structure and market power, this paper studies the relationship between the degree of vertical integration of coal enterprises and ICSR, which provides new empirical evidence and opens up a new way for the study of the strategic motivation of vertical integration of coal enterprises. In the new era of comprehensive social responsibility management in the coal industry, the conclusions of this paper can provide an important empirical basis for the formulation of supply-side reform and the optimization of incentive policies for fulfilling social responsibility in the energy structure optimization of the Chinese government. This paper can also help promote the clean and sustainable development of coal enterprises, ensure the fulfillment of social responsibility in the whole coal industry, and improve the mass emergence of technological innovation achievements.

### Study limitations

The research of this paper has the following limitations: First, there are many indicators for the vertical integration of enterprises, each with its own advantages and disadvantages, and the scholars have not yet reached a unified measurement standard. Considering that the coal enterprises are in the upstream of the industrial chain, and the vertical integration of some enterprises has made the upstream and downstream business become their main business and the inconvenience of dividing the main business and the auxiliary industry, the input-output table method proposed by Maddigan [57] is finally selected for calculation. Subsequent studies may consider optimizing the computational method of vertical integration and examining its impact on the results of the study. Second, the sample size is small. This paper selects the Shenzhen Stock Exchange and Shanghai Stock Exchange A-share listed coal enterprises for research, which can only represent a small part of the Chinese coal industry. We suggest that future research should use larger samples and include small and medium-sized coal enterprises in the sample, which may have different effects on the research results. Third, the chain intermediary role played by financing structure and market power is only a part of the intermediary. The other characteristics of the company must play a different role in the incentive of vertical integration to ICSR. Future research should pay attention to these issues.

## References

[1] Williams RH. Toward Zero Emissions from Coal in China. Energy Sustain Dev. 2001 5, 39–65. 10.1016/S0973-0826(08)60285-9

[2] National Statistical Bureau of the People’s Republic of China China Statistical Yearbook. Beijing:China Statistical Publishing House 2018.

[3] Zhao Y, Wang S, Duan L, Lei Y, Cao P, Hao J. Primary Air Pollutant Emissions of Coal-Fired Power Plants in China: Current Status and Future Prediction. Atmos Environ. 2008 42, 8442–8452. 10.1016/j.atmosenv.2008.08.021

[4] Bosch-Badia M.T, Montllor-Serrats J, Tarrazon-Rodon M.A. Corporate Social responsibility: a real options approach to the challenge of financial sustainability. Plos One. 2015,10, e0125972 10.1371/journal.pone.0125972 25938410 PMC4418608

[5] Zhao L, Gallagher K.S. Research, Development, Demonstration, and Early Deployment Policies for Advanced-coal Technology in China. Energy Policy. 2007 35, 6467–6477. 10.1016/j.enpol.2007.08.017

[6] Kong Y.S, Khan R. To examine environmental pollution by economic growth and their impact in an environmental Kuznets curve (EKC) among developed and developing countries. Plos One. 2019,14, e0209532 10.1371/journal.pone.0209532 30913276 PMC6435177

[7] Coase R.H. The Nature of the Firm. Economica. 1937 4, 386–405. 10.1111/j.1468-0335.1937.tb00002.x

[8] Sun Z, Zhu Y, Li Y, Xie M, Li G, Song J. Machine Learning Based Control Rights Analysis of Critical Resources and the Optimal Ownership for Management Integration. Cluster Comput. 2016 19, 1–11. 10.1007/s10586-016-0660-z

[9] Clarkson K.W, Miller L.R. Industrial Organization: Theory, Evidence, and Public Policy. Auckland: McGraw-Hill 1983.

[10] Hill C, Jones G, Schilling M.A. Strategic Management Theory: An Integrated Approach. USA: Houghton Mifflin 2001.

[11] Williamson Oliver E. Markets and Hierarchies: Analysis and Antitrust Implications. New York:Free Press 1975.

[12] Grossman S.J, Hart O.D. The Costs and Benefits of Ownership: A Theory of Vertical and Lateral Integration. J Polit Econ. 1986 94, 691–719. 10.1086/261404

[13] Hart O, Tirole J, Carlton D.W., Williamson O.E. Vertical Integration and Market Foreclosure. Brookings Papers on Economic Activity. Microeconomics. 1990 1990: 205–286. 10.2307/2534783

[14] Carlton D.W. Vertical Antegration in Competitive Markets under Uncertainty. J Ind Econ. 1976 27, 189–209. 10.2307/2098317

[15] Stigler G.J. Two Notes on the Coase Theorem. Yale Law J. 1989 99, 631–633. 10.2307/796757

[16] Porter M.E, Millar V.E. How Information Gives you Competitive Advantage. Harv Bus Rev. 1985 63, 149–174.

[17] Milgrom P, Roberts J. The Economics of Modern Manufacturing: Technology, Strategy, and Organization. Am Econ Rev. 1990 80, 511–528.

[18] Iversen T, Cusack T.R. The Causes of Welfare State Expansion: Deindustrialization or Globalization. World Polit. 2000 52, 313–349. 10.1017/S0043887100016567

[19] Frankel M. Obsolescence and Technological Change in a Maturing Economy. Am Econ Rev. 1955 45, 296–319.

[20] Kindleberger C.P. Economic Growth in France and Britain. USA:Harvard University Press 1964 1851–1950.

[21] Banerjee S, LIN P. Downstream R&D, Raising Rivals’ Costs, and Input Price Contracts. Int J Ind Organ. 2003 21, 79–96. 10.1016/S0167-7187(02)00010-3

[22] Buehler S, Schmutzler A. Intimidating Competitors—Endogenous Vertical Integration and Downstream Investment in Successive Oligopoly. Int J Ind Organ. 2008 26, 247–265. 10.1016/j.ijindorg.2006.11.005

[23] Lahiri N, Narayanan S. Vertical Integration, Innovation, and Alliance Portfolio Size: Implications for Firm Performance. Strateg Manag J. 2013 34, 1042–1064. 10.1002/smj.2045

[24] Karantininis K, Sauer J, Furtan W.H. Innovation and Integration in the Agri-Food Industry. Food Policy. 2010 35, 112–120. 10.1016/j.foodpol.2009.10.003

[25] Kapoor R, Adner R. What Firms Make VS. What They Know: How Firms’ Production and Knowledge Boundaries Affect Competitive Advantage in the Face of Technological Change. Organ Sci. 2012 23, 1227–1248. 10.1287/orsc.1110.0686

[26] Langlois P.L, Robertson R.N. Innovation, Networks, and Vertical Integration. Res Policy. 1995 24, 543–562. 10.1016/S0048-7333(94)00786-1

[27] Schoeffler S, Bain J. Barriers to New Competition. J Marketing. 1957 21, 488 10.1177/002224295702100413

[28] Bougheas S. Internal VS External Financing of R&D. Small Bus Econ. 2004 22, 11–17. 10.1023/B:SBEJ.0000011569.79252.e5

[29] Barney J.B, Hansen M.H. Trustworthiness as a Source of Competitive Advantage. Strateg Manag J. 1994 15, 175–190. 10.1002/smj.4250150912

[30] Dewaelheyns N, Hulle C.V. Internal capital markets and capital structure: bank versus internal debt. Eur Financ Manag. 2010 16, 345–373. 10.1111/j.1468-036X.2008.00457.x

[31] Twairesh A.E.M. The impact of capital structure on firm’s performance evidence from Saudi Arabia. J Appl Bank Financ. 2011 3, 183–193.

[32] Titman S, Wessels R. The determinants of capital structure choice. J Financ. 1988 43, 1–19. 10.1111/j.1540-6261.1988.tb02585.x

[33] Booth L, Aivazian V, Demirguc-Kunt A, et al Capital structures in developing countries. J Finan. 2001 56, 87–130. 10.1111/0022-1082.00320

[34] Frank M.Z, Goyal V.K. Testing the pecking order theory of capital structure. J Financ Econ. 2003 67, 217–248. 10.1016/S0304-405X(02)00252-0

[35] Chen J, Strange R. The determinants of capital structure: evidence from Chinese listed companies. Econ Change Restruct. 2005 38, 11–35. 10.1007/s10644-005-4521-7

[36] Morck R, Shleifer A, Vishny R.W. Management ownership and market valuation: an empirical analysis. J Financ Econ. 1988 20, 293–315. 10.1016/0304-405X(88)90048-7

[37] Goto A, Koga T, Suzuki K. The determinants of R&D investment in the Japanese manufacturing industries: small firms and large firms. Keizai Kenkyu. 2002 53, 18–23.

[38] Tamaschke R, Docwra G, Stillman R. Measuring Market Power in Electricity Generation: A long-term Perspective Psing a Programming Model. Energy Econ. 2005 27, 317–335. 10.1016/j.eneco.2005.01.001

[39] Klein B, Crawford R.G, Alchian A.A. Vertical integration, appropriable rents, and the competitive contracting process. J Law Econ. 1978 21, 297–326. 10.1086/466922

[40] Porter M.E. Competitive strategy. New York: The Free Press 1980.

[41] Blundell R, Griffiths R, Van Reenen J. Market share, market value and innovation in a panel of British manufacturing firms. Rev Econ Stud. 1999 66, 529–554. 10.1111/1467-937X.00097

[42] Ardianty F.D, Viverita D. Market power, efficiency and performance of Indonesian banks. Social Science Electronic Publishing. 2011.

[43] Zeng S.X, Xu X.D, Dong Z.Y, et al Towards corporate environmental information disclosure: an empirical study in China. J Clean Prod. 2010 18, 1142–1148. 10.1016/j.jclepro.2010.04.005

[44] Wang X.L, Fan G, Yu J.W. Market index report of China’s provinces (2016). Social Sciences Academic Press(China).2017,214–224.

[45] Wu J, Wang Q, Liu R. Marketization, FDI and the Technological Innovation of Domestic Enterprises—Based on the Method of Quintile. IEEE International Conference on Industrial Engineering & Engineering Management.2011.

[46] Gong G.M, Juan L. Evolvement of marketization, corporate governance and corporate performance: an empirical test of Chinese listed company based on the data of 2000-2009. IEEE International Conference on Intelligent System Design & Engineering Applications.2013.

[47] Fagerberg J, Mowery D.C, Nelson R. The Oxford Handbook of Innovation. UK:Oxford University Press 2005.

[48] Adelman M.A. The Concept and Statistical Measurement of Vertical Integration. Bus Concent Price Policy. 1955 6, 281–322

[49] Gort M. Diversification and Integration in American industry. USA:Princeton University Press 1962 16–19.

[50] Stucky J.A. Vertical Integration and Joint Ventures in the Aluminum Industry. USA:Harvard University Press 1983.

[51] Davies S, Morris W. ANew Index of Vertical Integration: Some Estimates for UK Manufacturing. Int J Indl Organ. 1995 13, 128–140.

[52] Spierdijka L, Zaourasa M. Measuring Banks’ Market Power in the Presence of Economies of Scale: A Scale-Corrected Lerner Index. J Bank Financ. 2018 87, 40–48. 10.1016/j.jbankfin.2017.09.022

[53] Baron R.M, Kenny D.A. The Moderator-Mediator Variable Distinction in Social Psychological Research: Conceptual, Strategic, and Statistical Considerations. J Pers Soc Psychol. 1986 51, 1173–1182. 3806354 10.1037//0022-3514.51.6.1173

[54] Preacher K, Hayes A. F. SPSS and SAS Procedures for Estimating Indirect Effects in Simple Mediation Models. Behav Res Methods Instrum Comput. 2004 36, 717–731. 10.3758/BF03206553 15641418

[55] Wu W, Liu Y, Chin T, Zhu W. Will Green CSR Enhance Innovation? A Perspective of Public Visibility and Firm Transparency. Int. J. Environ. Res. Public Health. 2018 15, 268 10.3390/ijerph15020268 PMC5858337 29401714

[56] Wang X, Lai W, Song X, Lu C. Implementation Efficiency of Corporate Social Responsibility in the Construction Industry: A China Study. Int. J. Environ. Res. Public Health. 2018 15, 2008 10.3390/ijerph15092008 PMC6165355 30223504

[57] Maddigan R.J. The Measurement of Vertical Integration. Rev Econ Stat. 1981 63, 328–335. 10.2307/1924349

